# Money matters (especially if you are good at math): Numeracy, verbal intelligence, education, and income in satisfaction judgments

**DOI:** 10.1371/journal.pone.0259331

**Published:** 2021-11-24

**Authors:** Pär Bjälkebring, Ellen Peters

**Affiliations:** 1 Department of Psychology, University of Gothenburg, Gothenburg, Sweden; 2 Center for Science Communication Research, School of Journalism and Communication, University of Oregon, Eugene, Oregon, United States of America; University of Edinburgh, UNITED KINGDOM

## Abstract

Objective numeracy, the ability to understand and use mathematical concepts, has been related to superior decisions and life outcomes. Unknown is whether it relates to greater satisfaction in life. We investigated numeracy’s relations with income satisfaction and overall life satisfaction in a diverse sample of 5,525 American adults. First, more numerate individuals had higher incomes; for every one point higher on the eight-item numeracy test, individuals reported $4,062 more in annual income, controlling for education and verbal intelligence. Combined, numeracy, education, and verbal intelligence explained 25% of the variance in income while Big-5 personality traits explained less than 4%. Further, the higher incomes associated with greater numeracy were related to more positive life evaluations (income and life satisfaction). Second, extant research also has indicated that the highly numerate compare numbers more than the less numerate. Consistent with numeracy-related income comparisons, numeracy moderated the relation between income and life evaluations, meaning that the same income was valued differently by those better and worse at math. Specifically, among those with lower incomes, the highly numerate were less satisfied than the less numerate; this effect reversed among those with higher incomes as if the highly numerate were aware of and made comparisons to others’ incomes. Further, no clear income satiation point was seen among those highest in numeracy, and satiation among the least numerate appeared to occur at a point below $50,000. Third, both education and verbal intelligence related to income evaluations in similar ways, and numeracy’s relations held when controlling for these other relations. Although causal claims cannot be made from cross-sectional data, these novel results indicate that numeracy may be an important factor underlying life evaluations and especially for evaluations concerning numbers such as incomes. Finally, this study adds to our understanding of education and intelligence effects in life satisfaction and happiness.

“Contentment makes poor men rich; discontent makes rich men poor.”

*Benjamin Franklin*

## Introduction

People value and pursue greater life satisfaction for themselves and others. As a result, understanding its antecedents is important because it may help individuals and organizations focus on actions more likely to lead to long-term increases in life satisfaction [1]. Although education has been linked with higher life satisfaction [2], reasons for its connection remain unclear.

Classic economic theory suggests that intelligence (“reason”) produces better decisions, more wealth, and higher levels of “joy” and life satisfaction [3]. Conventional wisdom informs us that the opposite can be true–“ignorance is bliss.” That is, being smart comes with a price, and that price is reduced life satisfaction (think about the tortured genius and socially excluded nerd). Some theories support this conventional wisdom, including those positing that individuals with high cognitive ability react with over-excitable emotional and behavioral responses to their environment. Mood disorders, for example, are more than three times as frequent among those with an IQ of 130 or above, compared to the general population [4].

Further, formal education arguably can increase intelligence [5,6]. However, intelligence is a multifaceted construct of cognitive abilities, meaning that people can be intelligent in different ways [7]. And it is unclear what kinds of intelligence might relate to life satisfaction specifically. For example, research has pointed towards education’s positive effects on some life outcomes as being due to numeric intelligence [8,9] (aka, objective numeracy, defined as the ability to understand and use probabilistic and other mathematical concepts). In the present paper, we focus on numeric intelligence, controlling for education and verbal intelligence [10]. Numeric intelligence is potentially important because numbers instruct, inform, and give meaning to information intended to improve everyday judgments and choices, and those with greater numeracy generally understand more numeric information and make superior judgments and choices when numbers are involved [11–13].

Consistent with making better decisions, the more objectively numerate report more positive financial outcomes. For example, more numerate individuals have higher incomes and are less likely to be unemployed long-term (independent of education and literacy [14,15]). In an English sample of individuals over 50 years, highly numerate people had more wealth compared to the less numerate, controlling for education and cognitive abilities such as literacy and executive function [16]. Together, these findings suggest that objective numeracy is important for financial outcomes.

Additionally, income is often studied as a major driver of overall life satisfaction [17]. People in high income nations are happier, on average, than those in very poor nations, just as people with higher incomes within a nation tend to be happier than those with smaller incomes [18]. Income’s relation with life satisfaction also appears causal; a medium-sized lottery win produced increased life satisfaction two years later [19]. We hypothesized:

Hypothesis 1 (H1): Greater objective numeracy would be associated with greater income satisfaction and greater life satisfaction through its relation with higher income (controlling for education, verbal intelligence, and personality).

Inconsistent with this hypothesis, however, average happiness has remained constant over the last decades while income has grown exponentially [20]. This inconsistency may be due to greater income leading to higher expectations [21] and/or aspirations [22]; these higher expectations and aspirations, in turn, may dampen satisfaction if they go unmet. Another common explanation for this paradox is that people do not judge happiness based on their absolute income. Instead, they evaluate it based on their income relative to others’ income [23]. If true, the more educated should experience greater life satisfaction (and they do [24]) if for no other reason than they have higher average incomes. However, those with high education and lower incomes (compared to other highly educated people) are less satisfied with their lives than those with lower education and the same income [24]. Although the mechanisms are unknown for this link between higher education, lower income, and less satisfaction, it has been hypothesized to involve numeric computations and comparisons [25].

However, people differ in their tendencies to perform and rely on numeric comparisons. In particular, more numerate individuals are more inclined than the less numerate to compare numbers, presumably due to more accessible number operations [10,26,27]. Even when the less numerate are capable of similar comparisons, they appear to need more direction or motivation to do so [28,29]. Hence, we reasoned that the highly numerate would be more likely than the less numerate to compare incomes so that they were more income-sensitive in their satisfaction ratings, even after controlling for other factors.

Hypothesis 2 (H2): Numeracy would moderate the relation between income and satisfaction such that the highly numerate would be more satisfied than the less numerate at higher income levels but less satisfied at lower incomes (controlling for education, verbal intelligence, and personality).

The present study explores these hypotheses concerning the role of numeracy in income satisfaction and life satisfaction using a diverse American sample. The study breaks new ground in that it is the first study of which we are aware to connect a specific cognitive ability (numeracy, controlling for education and a non-numeric intelligence) to more general ratings of one’s life (i.e., income satisfaction and life satisfaction). The study examines numeracy as a potentially important cognitive factor underlying how people evaluate their lives, with a particular focus on a number-comparison inclination for the highly numerate that has been found in prior well-controlled lab studies.

## Method

The project described in this paper relies on data from surveys administered by the Understanding America Study (UAS), which is maintained by the Center for Economic and Social Research at the University of Southern California (USC). Detailed methodology information is available at uasdata.usc.edu. All procedures, including the informed consent process, were conducted in accordance with the ethical standards of the responsible committee on human experimentation (institutional and national) and with the Helsinki Declaration of 1975, as revised in 2000. UAS panel procedures have been approved by the USC Institutional Review Board (IRB). Both data and syntax are available for interested researchers who want to explore them in more detail. Data are openly available at https://uasdata.usc.edu/ and syntax for the present analyses is available from https://osf.io/uwq7v/; all variables are part of the UAS Comprehensive File that can be accessed after registration at https://uasdata.usc.edu/page/UAS+Comprehensive+File. The authors had no special access privileges to the data.

### Participants

Participants were internet panel members from the UAS. All participants were recruited using an address-based sampling method. USC provided Internet access to participants who did not have it. Panel members completed surveys and were paid for each completed questionnaire (e.g., $5 for a 5-minute survey). The UAS, which began in 2014, represents one of the richest sources of panel data available in the United States (see Alattar, Messel, & Rogofsky [30] for an overview). For this study, we compiled data across three survey modules (Modules 1, 2, and 44). Participants who had completed all three modules by April 26^th^, 2018 were included in our study (N = 5,748). Only participants answering all relevant measures were included in analyses, leaving a final N = 5,525. Based on 2010 U.S. Census records, our sample was more educated (Bachelor’s degree or more = 35.9% vs. 27.2%), older (median age = 48 years vs. 37 years) and included more women (57.1% vs. 50.9%) relative to the U.S. population.

### Measures

Age was treated as a continuous variable using the participant’s age in whole years, in addition we included an age^2^ term to account for nonlinear relations between age and the outcomes (age was divided by 10 and age^2^ was divided by 100 to increase readability of betas and confidence intervals in regressions models) [31,32]. Gender was recoded (0 = female; 1 = male). Education was measured on a 16-point scale from 1 = 1st grade through 16 = Doctorate degree. However, to establish a normal distribution, and for consistency with similar studies exploring education effects [33], responses were categorized: 1 = Less than High School diploma, 2 = High school diploma, 3 = Some college or Associates degree, 4 = Bachelor’s degree, and 5 = Master’s degree or more. Participants answered their household income range on a 16-point scale from 1 = $0-$5,000 through 16 = $150,000 or more. This scale was converted into dollars by using the midpoint of the range. Further, to account for diminishing marginal utilities of income in line with Kahneman and Deaton [17], the logarithm of income (log_10_ income) was used in the main regressions (unless otherwise mentioned).

To assess objective numeracy, participants completed a traditional eight-item numeracy scale [34] (e.g., “If the chance of getting a disease is 10%, how many people would be expected to get the disease out of 1,000?”). Each item was scored as correct or incorrect, and correct items were summed (possible range 0 to 8). Missing responses were coded as incorrect.

Covariates were assessed. For non-numeric intelligence, participants completed 15 verbal intelligence problems (verbal logic: e.g., “Mother is to Daughter as Father is to ____”). Each item was scored as correct or incorrect, and correct items were summed (possible range 0 to 15). Missing responses were coded as incorrect. Such verbal analogies have long been viewed as a general-intelligence measure [35]. Further, because personality also relates to both life outcomes and life satisfaction [36], we controlled for Big-Five personality factors [26] in all analyses. Participants responded to 44 questions measuring Extraversion (8 items), Agreeableness (9 items), Conscientiousness (9 items), Neuroticism (8 items), and Openness (10 items) on a scale from 1 = Disagree Strongly to 5 = Agree strongly.

Income and life satisfaction were measured with one-item questions on scales from 0 = not at all to 10 = completely (“Overall, how satisfied are you with your income?” and “Overall, how satisfied are you with your life?”).

### Analysis approach

To test our two hypotheses, we used separate regression analyses for income satisfaction and life satisfaction. We regressed each satisfaction variable onto objective numeracy, verbal intelligence, Big-Five personality factors, and demographics (age, age^2^, gender, education, and income). We note that our analytical approach treats education as a covariate even though education likely has bidirectional causality with both objective numeracy and verbal intelligence. Relations between these variables, thus, are simplified in the present paper.

**Fig 1:**
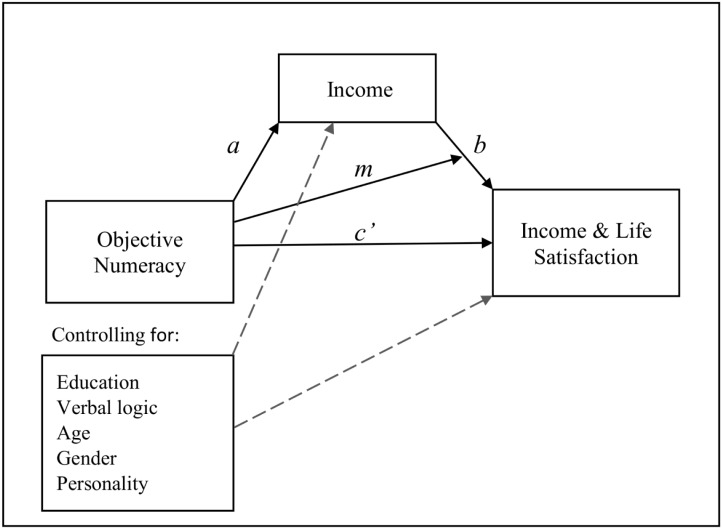
Hypothesized suggesting that objective numeracy has both a moderating effect and an indirect effect on satisfaction through the relation between income and satisfaction. *a* represents the path between objective numeracy and income. *c’* represents the residual direct effect of objective numeracy on satisfaction, while controlling for all covariates including income. *b* represents the direct effect of income on satisfaction, while controlling for the moderating effect of objective numeracy on *b*. *m* represents the moderating effect of objective numeracy on the relation between income and both income and life satisfaction.

All regression models were performed in R-statistics (version 4.0.4). Continuous predictors were mean centered, so that models were centered at the average level of these predictors. Standardized estimates (betas) were calculated for continuous variables. For gender, a partially standardized estimate was calculated that represented the difference in standard deviations in the dependent variable between men and women. We used the Lavaan package in R [37] to estimate indirect effects. Hence, and as depicted in Fig 1, we estimated (*a*) the direct effect of objective numeracy on income and (*c*’) the residual direct effect of objective numeracy on income satisfaction and life satisfaction, while controlling for all covariates including income. We also estimated (*b*) the direct effect of income on both satisfaction variables and (a*b) the indirect effect of objective numeracy on both satisfaction variables through income, while controlling for (*m*) the moderating effect of objective numeracy on the paths between income and both income satisfaction and life satisfaction.

Lavaan allow for indirect effects to be estimated through bootstrapped confidence intervals, following recommendations from Preacher and Hayes [38]. Further and in accordance with Kenny and Judd [39], we will avoid making claims about full or partial mediation as the power to detect the residual direct effect is much lower than the power to detect the indirect effect. Thus, our results are focused on the indirect effect (H1) as well as the moderating effect (H2) of objective numeracy on income and life satisfaction. Lastly, we will focus on results related to our hypotheses. Thus, results related to covariates will not be discussed in detail although they can be seen in Tables 1–3 and supporting information S1–S6 Tables. All this said, this study relies on cross sectional data from which causation cannot be proven. However, the models in this study are theory-based and test plausible correlational models. They therefore suggest causal models worthy of further investigation.

**Table 1:** Means, standard deviations, and ranges for continuous measures and Cronbach’s alpha for multi-item indexes.

	Mean	SD	Min	Max	*α*
Independent measures					
Objective numeracy	3.6	1.9	0	8	.72
Age (years)	47.6	15.5	17	106	-
Income (thousands $)	62.4	43.2	2.5	150	-
Education	3.2	1.1	1	5	-
Verbal Intelligence	12.1	2.6	0	15	.78
Extraversion	3.3	.8	1	5	.81
Agreeableness	4.0	.6	1	5	.75
Conscientiousness	4.1	.6	1	5	.77
Neuroticism	2.7	.8	1	5	.82
Openness	3.6	.8	1	5	.77
Dependent measures					
Income Satisfaction	5.6	2.7	0	10	-
Life Satisfaction	7.3	1.9	0	10	-

**Table 2:** Linear regression analysis results of income predicted from objective numeracy, verbal intelligence, education, gender, age, age^2^, and Big-Five personality factors.

	Income			
	*beta*	*b*	95% CI [LL, UL]	*p*
Intercept		3.81	[3.77, 3.84]	< .001
Objective Numeracy	.18	0.09	[0.08, 0.11]	< .001
Covariates				
Verbal intelligence	0.15	0.06	[0.05, 0.07]	< .001
Education	0.29	0.27	[0.25, 0.29]	< .001
Gender	0.15	0.15	[0.11, 0.20]	< .001
Age	0.09	0.06	[0.05, 0.08]	< .001
Age^2^	-0.11	-0.04	[-0.05, -0.03]	< .001
Extraversion	0.08	0.10	[0.07, 0.13]	< .001
Agreeableness	-0.03	-0.05	[-0.09, -0.01]	.021
Conscientiousness	0.07	0.11	[0.07, 0.15]	< .001
Neuroticism	-0.04	-0.05	[-0.08, -0.02]	.003
Openness	-0.10	-0.17	[-0.20, -0.13]	< .001

*Note*. *Beta* indicates the standardized regression coefficient (partially standardized for Gender; 0 = female; 1 = male). *b* represents unstandardized regression weights. *LL* and *UL*, respectively, indicate the lower and upper limits of a confidence interval around the *b*. Model R^2^ = .31, F(11,5513) = 223.5, *p* < .001, adjusted R^2^ = .31, AIC = 13404, BIC = 13490.

**Table 3:** Linear regression analysis results of income satisfaction and life satisfaction predicted from income, objective numeracy, their interaction, verbal intelligence, education, gender, age, age^2^, and Big-Five personality factors.

	Income Satisfaction					Life Satisfaction			
	*beta*	*b*	95% CI [LL, UL]	*p*	*—*	*beta*	*b*	95% CI [LL, UL]	*p*
Intercept		5.27	[5.17, 5.37]	< .001			7.13	[7.06, 7.21]	< .001
Income (log_10_)	.42	2.67	[2.49, 2.85]	< .001		0.26	1.13	[0.99, 1.26]	< .001
Objective Numeracy	.02	0.02	[-0.02, 0.06]	.260		-0.03	-0.03	[-0.06, 0.00]	.064
Income (log_10_) x Objective numeracy	.10	0.33	[0.26, 0.41]	< .001		0.08	0.19	[0.13, 0.25]	< .001
Covariates									
Verbal intelligence	-.01	-0.01	[-0.04, 0.02]	.369		-0.03	-0.02	[-0.04, 0.00]	.068
Education	.03	0.06	[-0.00, 0.13]	.059		-0.02	-0.03	[-0.08, 0.02]	.205
Gender	-.01	-0.02	[-0.15, 0.12]	.805		-0.06	-0.10	[-0.20, -0.00]	.040
Age	.07	0.13	[0.09, 0.17]	< .001		0.02	0.02	[-0.01, 0.05]	.011
Age^2^	.08	0.08	[0.06, 0.10]	< .001		0.09	0.06	[0.05, 0.08]	< .001
Extraversion	.04	0.12	[0.03, 0.20]	.006		0.09	0.22	[0.15, 0.28]	< .001
Agreeableness	-.00	-0.02	[-0.14, 0.10]	.732		0.03	0.08	[-0.00, 0.17]	.055
Conscientiousness	.04	0.19	[0.08, 0.31]	.001		0.06	0.20	[0.11, 0.28]	< .001
Neuroticism	-.16	-0.51	[-0.60, -0.42]	< .001		-0.27	-0.63	[-0.69, -0.56]	< .001
Openness	-.10	-0.42	[-0.53, -0.32]	< .001		-0.08	-0.25	[-0.33, -0.17]	< .001
Model Fit									
Adjusted R^2^	.25					.19			
AIC	24929					21507			
BIC	25029					21606			

*Note*. *beta* indicates the standardized regression coefficient (partially standardized for Gender; 0 = female; 1 = male). *b* represents unstandardized regression coefficient. *LL* and *UL* respectively indicate the lower and upper limits of a confidence interval around *b*.

## Results

### Sample characteristics, reliability, and simple correlations

Descriptive statistics are presented in Table 1. As expected, more numerate individuals had higher income (*r* = .39, *p* < .001), more education (*r* = .42, *p* < .001), and greater verbal intelligence (*r* = .50, *p* < .001). In addition, men (43% men, 57% women) were more numerate than women (respective means = 4.14 and 3.15, *t*(5135.7) = 19.99, *p* < .001). Numeracy was also related to greater income satisfaction (*r* = .18, *p* < .001) and, to a much lesser degree, greater life satisfaction (*r* = .03, *p* = .011; see supporting information S1 Table for correlations among all variables).

#### Testing H1 and H2

First, we confirmed that numeracy indeed was related to higher income (a-path in Fig 1) in this large, diverse U.S. sample, controlling for verbal intelligence, education, gender, age, age^2^, and Big-Five personality factors (see Table 2). The best predictors of income were education (beta = .29), objective numeracy (beta = .18), and verbal intelligence (beta = .15). Together, these three predictors explained 25% of the variance in income whereas the Big-Five personality factors together explained only 3.4%. These income effects were not small. For every one point higher on the eight-item numeracy test, annual income was higher by $4,062 (see supporting information S2 Table for full regression results using non-logged income). The average annual income difference between participants scoring the lowest vs. highest on the numeracy scale was about $36,000, controlling for education, verbal intelligence, age, gender, and personality.

Next, to test our two hypotheses, we conducted separate linear regressions of income satisfaction (R^2^ = .25, F(13,5511) = 140.4, *p* < .001) and life satisfaction (R^2^ = .19, F(13,5511) = 100.8, *p* < .001) using predictors of income, objective numeracy, and their interaction, controlling for verbal intelligence, education, gender, age, age^2^, and Big-Five personality factors. See Table 3 for the full results.

To test H1 (that greater objective numeracy would be associated with greater income satisfaction and greater life satisfaction through its relation with higher income), we estimated the indirect effects of numeracy through income on the satisfaction variables. The analysis confirmed that the relation of numeracy with income satisfaction was mediated through income (indirect effect 0.11, 95% CI [0.09, 0.13], p < .001); similar results emerged for life satisfaction (indirect effect 0.04, 95% CI [0.03, 0.05], p < .001). Thus, H1 was supported. Greater objective numeracy was associated with having more income which, in turn, related to greater income satisfaction and life satisfaction.

**Fig 2:**
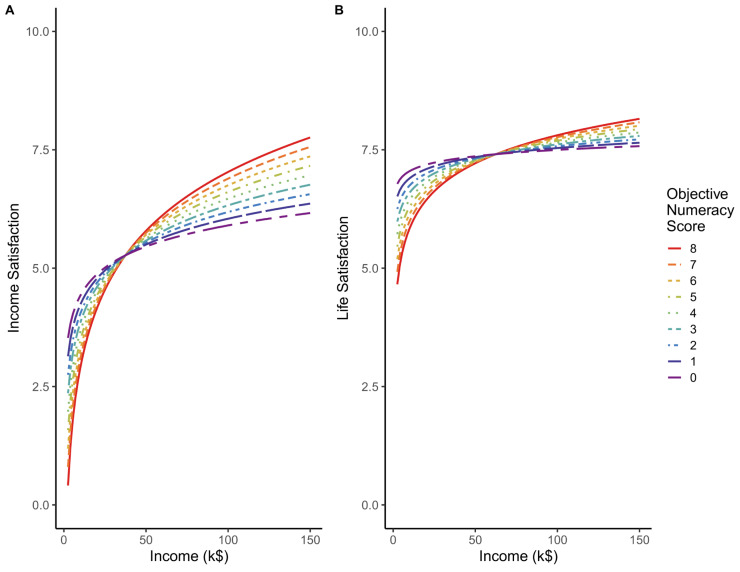
Estimated means of income satisfaction (panel A) and life satisfaction (panel B), plotted as a function of income (thousands of $) and objective numeracy (percentile). The graph indicates that the relation between income and satisfaction is stronger in those with higher numeracy compared to those lower in numeracy, for both income satisfaction and life satisfaction. Thus, numeracy seems to be an important factor when predicting the point of income satiation at the individual level.

To test H2 (that numeracy would moderate the relation between income and satisfaction, indicating that income had different effects for the more and less numerate), we examined the interaction of objective numeracy and income in both analyses. As indicated in Table 3, the interaction was significant for income satisfaction (interaction *beta =* .*10*, *b* = 0.33, *p* < .001; see Fig 2A for plotted relation). Analyses of life satisfaction were similar (interaction *beta* = .08, *b* = 0.19, *p* < .001; see Fig 2B for plotted relation). Thus, higher numeracy was associated with higher incomes (Table 2), and also those higher in numeracy evaluated their higher incomes more positively (Table 3).

**Fig 3:**
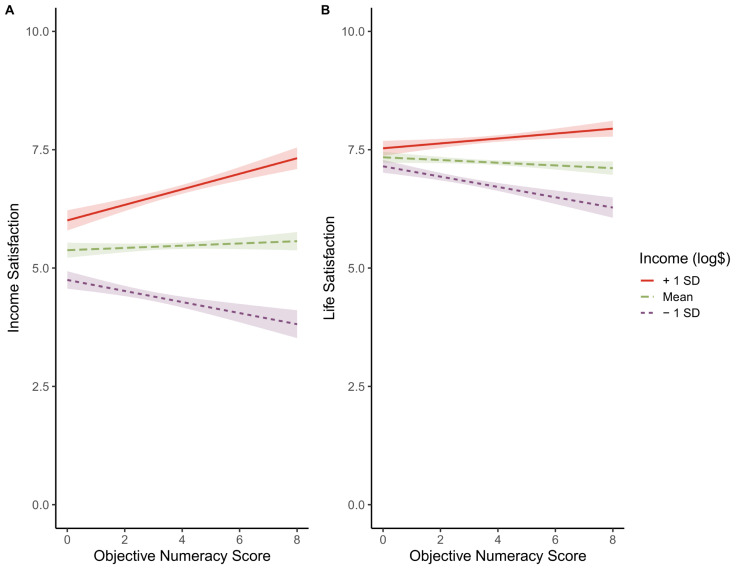
Estimated means and 95% confidence intervals of income satisfaction (panel a) and life satisfaction (panel b), plotted as a function of objective numeracy (0–8) and income (logged $). The graphs indicate that people higher in numeracy had higher satisfaction than those lower in numeracy if their income was higher (the top line) and had lower satisfaction if their income was lower (the bottom line), for both income satisfaction and life satisfaction. Thus, those who scored highest on the numeracy test had either the absolute highest or the absolute lowest average levels of income satisfaction and life satisfaction, depending on their income.

Simple slopes suggest that greater objective numeracy related to higher income satisfaction at higher income levels (*b*_*+1 SDIncome*_ = 0.16, 95% CI [0.10, 0.22], *p* < .001). However, at lower income levels, being more numerate was related to lower income satisfaction (*b*_*-1 SDIncome*_ = -0.12, 95% CI [-0.18, -0.06], *p* < .001); see Fig 3A. Similar results emerged for life satisfaction (*b*_*+1SD Income*_ = 0.5, 95% CI [0.01, 0.09], *p* = .01; *b*_*-1SD Income*_ = -0.11, 95% CI [-0.15, -0.07], *p* < .001); see Fig 3B.

Furthermore, the data were a better fit to the two models that included the numeracy-income interaction than those that did not include it (income satisfaction: ΔR^2^ = .8%, F(1,5512) = 73.8, *p* < .001; life satisfaction: ΔR^2^ = .7%0, F(1,5512) = 44.9, *p* < .001). For full regression results when the interaction was not included, see supporting information S4 Table.

**Fig 4:**
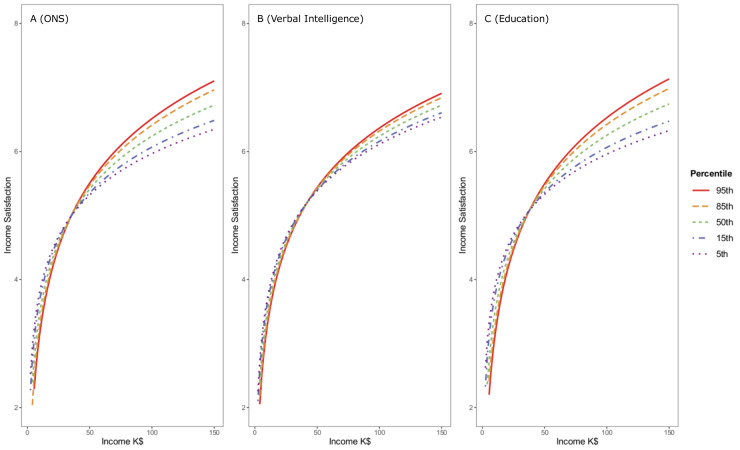
Estimated means of income satisfaction plotted as a function of income (thousands of $) and objective numeracy (panel A), Verbal Intelligence (panel B), and Education (panel C). Each moderator added significant predictive power to the model, meaning that each one altered the predictive power of income on income satisfaction, independent of the others.

**Fig 5:**
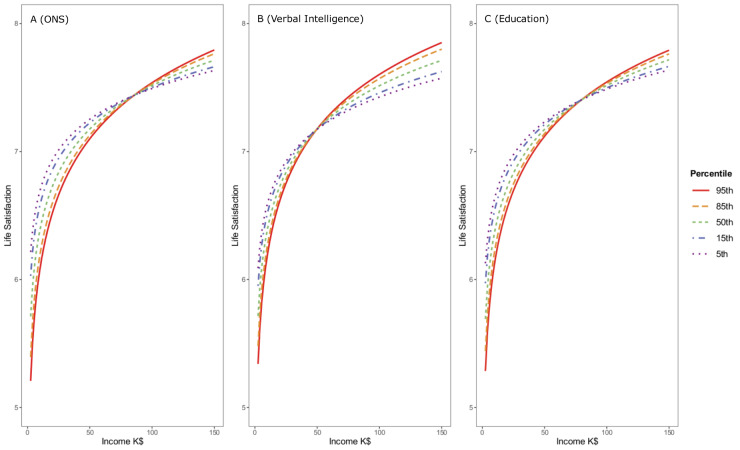
Estimated means of life satisfaction plotted as a function of income (thousands of $) and objective numeracy (panel A) Verbal Intelligence (panel B) and Education (panel C). Each of the moderators remained significant in the model, meaning that each moderator altered the association of income with income satisfaction.

Further, to examine the robustness of the numeracy moderation, we fit the data to models of each satisfaction variable in which education, verbal intelligence and numeracy all moderated income’s effect on satisfaction. We also included household size in this robustness check and restricted the sample to only working age adults (those in our sample 65 years or younger), as income in retirement might be evaluated differently especially for those higher in numeracy. This left us with a sample of N = 4,574 American adults age 65 years or younger. As hypothesized, the moderating effect of numeracy remained significant, albeit descriptively smaller, in this smaller sample. Further, the moderating effect of numeracy was comparable to the moderating effects of both education and verbal intelligence in models of both income satisfaction (numeracy*income *beta* = .06, *p* < .001, education*income *beta* = .07, *p* < .001, and verbal intelligence*income *beta* = .03, *p* = .017) and life satisfaction (numeracy*income *beta* = .05, *p* = .007, education*income *beta* = .04, *p* = .012, and verbal intelligence*income *beta* = .04, *p* = .006). Hence, as suggested in previous studies, education appears to exert its own influence, in interaction with income, on both income satisfaction and life satisfaction. However, numeracy has an independent relation, that is also independent of possible effects of verbal intelligence; see supporting information S5 Table for the full results of the model and see Figs 4 and 5 for the plotted relationships. In a final analysis aimed at evaluating the robustness of our findings, we examined these same models (as in the S5 Table), but treated education as a factor instead of assuming linearity of its relations. Again, numeracy moderated the relation of income with both income satisfaction (numeracy*income *b* = .18, *p* < .001) and life satisfaction (numeracy*income *b* = .12, *p* = .002). For full results, see supporting information S6 Table.

To sum up, our data suggest that objective numeracy had an indirect effect on life evaluations through income, and it moderated the relation of income for both satisfaction variables. These data support our supposition that those higher and lower in objective numeracy used income differently to evaluate their lives, irrespective of their level of education or verbal intelligence.

## Discussion

The modern world is full of numbers: cash, calories, and credit scores. Not surprisingly, research has shown that those adept with numbers experience better financial outcomes [40,41]. The present study supports these findings and further demonstrates that numeracy related to life evaluations (i.e., income satisfaction and life satisfaction). First, objective numeracy had a significant and positive association with income; each additional correct answer on the eight-question numeracy test was associated with $4,062 more in yearly income, controlling for other individual differences such as education, personality, and verbal intelligence. Thus, although numeracy was related to these variables, it nonetheless was uniquely associated with income [5,6]. Further, objective numeracy was among the three best predictors of income (together with education and verbal intelligence). Hence, being intelligent, including numerically intelligent, appears to have positive financial consequences for individuals in the form of higher incomes [42].

We further demonstrated for the first time that numeracy had a significant indirect effect, through increased income, on both income satisfaction and life satisfaction. Hence, being good at math appeared to support the ability to make money, and, through this income, it predicted greater satisfaction with both income and life [17]. Although similar results have been shown for education [43], our results held while controlling for education and verbal intelligence. These results thus provide support for numeracy as a potential key cognitive factor for wellbeing. It further suggests that the cognitive skills learned through schooling, numeric skills and verbal skills, may exert separate influences on wellbeing [23–25].

In addition, we tested the novel hypothesis that the relative-income effect on life evaluations [25] would be moderated by numeracy. We know that more numerate people are more inclined naturally to do number comparisons than the less numerate [28]. We reasoned that this comparison habit should extend to income comparisons. In fact, numeracy moderated the effect of income on life evaluations. In a robustness check, this hypothesis was supported even after controlling for education, verbal intelligence, their respective interactions with income, and household size and after restricting the sample to working age adults and adding a quadratic age term to allow satisfaction to vary nonlinearly with age.

Overall, simple slopes designed to test our hypothesis indicated that income had little effect on satisfaction among the less numerate; they showed very little of the standard relative income effect [23]. However, income had strong relations with income satisfaction and life satisfaction among the highly numerate. In fact, at higher incomes, the highly numerate were more satisfied than the less numerate. This relation reversed at lower income levels. Ignorance can be bliss, and, as Benjamin Franklin opined, it may “make poor men rich.” These results and reasoning further imply that the ~$70,000 satiation point found in some past research will depend on individual differences, such as objective numeracy. In our data, no clear satiation point existed among those highest in numeracy, and satiation among the least numerate appeared to occur at a point below $50,000 (see Fig 2).

Thus, numeric ability may help in two ways. First, it appears to support the ability to attain a higher income [14,15]. Second, once the person earns more income, being more numerate supports the interpretation that all is good with the world. However, being more numerate is a double-edged sword as those highly numerate individuals with lower incomes were particularly dissatisfied. These relations held after controlling for education, verbal intelligence, age, gender, and Big-Five personality factors. Further, our data support earlier findings that the more educated are more satisfied when they have higher income rather than lower [24]. Nonetheless, numeracy had a separable and independent effect over and above education. Because numeracy and education are related, it is possible that previous studies may have conflated the effect of numeracy as being due to education. Parsing out these differences can help to inform about education’s relations with outcomes and also why those relations occur (e.g., through numeric ability) [23–25]. These numeracy findings point towards a specific potential role for numeric comparisons in how we judge our life situation that will vary by individual differences.

Of great interest to us, in a model where numeracy, education, and verbal intelligence all were allowed to moderate income’s influence, they all did so significantly. These findings can be interpreted either as all three effects being due to a confounding variable, perhaps socioeconomic status or general intelligence [5,7]. Alternatively, these effects may be explained by numeracy, education, and verbal intelligence all having separate psychological or other influences. For example, greater numeracy may relate to people making more income comparisons [25], whereas verbal intelligence and/or education may help people see new possibilities that lead to greater life expectations and therefore persistence [21]. Further studies are needed to pinpoint these processes.

Implications exist, too, for the numeracy literature. Although the highly numerate are more likely than the less numerate to compare numbers [10], the less numerate are capable of comparing numbers and can be motivated to do so [28]. Hence, the highly numerate appear to have number-processing habits (perhaps due to chronically greater access to numeracy knowledge structures [44]) that the less numerate do not share. These habits, in turn, appear to influence how they judge and decide. Although we cannot pinpoint how the process of income comparison differs between those with higher and lower numeracy at this time, several possible mechanisms exist. For example, the highly numerate may develop stronger feelings about their personal income by comparing it to those of others and then use this affect to guide evaluations [26]. Alternatively, the highly numerate may attend to, search for, or recall other incomes better [45]. It is also possible that the less numerate did compare their incomes, but to more proximal and familiar incomes among their family and friends whereas the highly numerate made comparisons to a broader income set. Such thinking is consistent with the highly numerate processing numeric problems more abstractly than the less numerate [46].The lack of a relative income effect among the less numerate, however, suggests that they may derive satisfaction using mechanisms other than income comparisons. Being able to recognize and use such a process would be helpful, perhaps particularly for the highly numerate with lower incomes. Further research is needed.

One important theoretical point is that improving a person’s numeracy likely will increase satisfaction only if it is accompanied with an above-average income; if accompanied with lower salaries, improved numeracy instead might lead to dissatisfaction [23]. Alternatively, one could argue that it would be better if people only compared incomes when it was beneficial to them; however, for the highly numerate, doing number comparisons seems almost automated [28]. If true, this speculation leaves open the possibility that increasing numeracy will pose a particular problem for groups who have historically suffered from income inequality, for example, women and minorities [47]. Thus, further exploration of the role of numeracy in income (and income-satisfaction) inequality is warranted.

The predictive power of objective numeracy is particularly interesting relative to personality factors, because numeracy can be improved in children and (with more effort) in adults [48,49]. Formal education is thought to increase numeracy and, through it, to improve decision abilities and life outcomes [50]. Recent research also has demonstrated that state-mandated high school mathematics courses (but not personal finance courses) led to greater investment income, better credit management, and fewer foreclosures [51]. Further, causal effects exist. A 9-week longitudinal study among college students enhanced objective numeracy and financial literacy, in turn [48]. Thus, the present results suggest that taking steps to improve objective numeracy may improve one’s life circumstances and satisfaction. In contrast, personality also has an association with both income and life satisfaction [52]. However, personality is generally thought to be a stable trait, and, hence, changing it has less potential for improving people’s lives.

The aim of this study was to investigate relations between income, objective numeracy, and satisfaction in a large, diverse sample of Americans [30]. The relations in these data fit our theory, and both hypotheses were supported. Nonetheless, we cannot make causal claims. Follow-up studies are needed using experimental manipulations of numeracy and/or a longitudinal design with multiple assessments of life satisfaction and objective numeracy. This latter study, while worthwhile, would take years and possibly decades to complete. A related possibility would be to engage with ongoing or past math-intervention studies and examine whether they improved numeric ability and later financial outcomes and life satisfaction.

We believe that being better at math is related to thinking about, interacting with, and viewing the world in new ways. Theoretical models of intelligence suggest that rational thinking emerges from two or even three subsystems [53]. Based on these models and our data, it is likely that objective numeracy captures several different psychological processes—some related to earning higher incomes (e.g., skills that lead to better paying jobs), others related to viewing one’s income differently (e.g., number comparisons) [54,55]. For instance the strong relative-income effect among the highly numerate may reflect what has been called “serial associative cognition” [53], a form of deliberative processing that is incomplete and fixated on a subset of information. Future studies should try to identify and separate these psychological processes. Hence, in-depth exploration is needed of why and when different cognitive abilities are important to improved outcomes and satisfaction [2]. The present research points to objective numeracy being especially important for life outcomes. However, further exploration also is warranted of other cognitive and motivational factors related to number use (e.g., numeric confidence and numeric magnitude mappings) and non-number use [10].

Moreover, we likely did not capture the upper or lower bounds of the numeracy-income relation [10]. We used a relatively brief eight-item numeracy scale, and each level was associated with large income gains. A more detailed investigation of boundary conditions (where the influence of numeracy tapers off) would make it possible to create a scale that better captures both upper and lower levels of “functional” numeracy (i.e., the upper and lower boundaries of numeracy within which being more or less numerate makes a difference) [34,45]. Finally, objective numeracy has been linked to life outcomes other than income [26,40,50]. Future studies should focus on health, wealth, and other resources that differ across individuals and across the lifespan. For example, wealth (and therefore perhaps numeracy) can buffer against declines in life satisfaction during stressful life events [56].

## Conclusion

In closing, these findings are consistent with the importance of numeric intelligence to income, income satisfaction, and life satisfaction [26]. The personal utility derived from income for income satisfaction and life satisfaction appears highly dependent on numeric intelligence, supporting the potential importance of numeric comparisons and the number-comparison inclinations of the highly numerate [28]. Lastly, the results point towards novel means by which people might improve their lives. In particular, because objective numeracy is an acquired skill, education (especially in math) may create objective and subjective benefits across the lifespan [48], however, the subjective benefits may accrue only to highly numerate people with higher incomes.

## Supporting information

S1 Table Correlations of predictors with income satisfaction and life satisfaction. (DOCX)

S2 Table Linear regression analysis results of income (in thousands of $) predicted from objective numeracy, verbal logic, education, gender, age, age^2^, and Big-Five personality factors. (DOCX)

S3 Table Regression analysis results of Income satisfaction and life satisfaction predicted from objective numeracy, verbal logic, education, gender, age, age^2^, and the Big-Five personality factors. (DOCX)

S4 Table Regression analysis results of Income satisfaction and life satisfaction predicted from income, objective numeracy, verbal logic, education, gender, age, age^2^, and the Big-Five personality factors. (DOCX)

S5 Table Regression analysis results of Income satisfaction and life satisfaction predicted from income, objective numeracy, verbal logic, education, gender, age, age^2^, and the Big-Five personality factors as well as the three interactions between income and objective numeracy, income and verbal logic and income and education. (DOCX)

S6 Table Regression analysis results of Income satisfaction and life satisfaction predicted from income, objective numeracy, verbal logic, education (as a factor), gender, age, age^2^, and the Big-Five personality factors as well as the three interactions between income and objective numeracy, income and verbal logic and income and education. (DOCX)
