# Supplementary material for: Money matters (especially if you are good at math): Numeracy, verbal intelligence, education, and income in satisfaction judgments
Source: PLoS One. 2021 Nov 24;16(11):e0259331. doi: 10.1371/journal.pone.0259331 (PMC8612560; doi:10.1371/journal.pone.0259331)
Supplement: S1 Table — (DOCX) [file pone.0259331.s001.docx]

# Table S1. *Correlations of predictors with income satisfaction and life satisfaction.*

|  | 1 | 2 | 3 | 4 | 5 | 6 | 7 | 8 | 9 | 10 | 11 | 12 |
| --- | --- | --- | --- | --- | --- | --- | --- | --- | --- | --- | --- | --- |
| 1. Objective Numeracy | - |  |  |  |  |  |  |  |  |  |  |  |
| 2. Verbal logic | .50** | - |  |  |  |  |  |  |  |  |  |  |
| 3. Education | .42** | .33** | - |  |  |  |  |  |  |  |  |  |
| 4. Gender | .26** | .09** | .04** | - |  |  |  |  |  |  |  |  |
| 5. Age | -.00 | -.01 | .06** | .14** | - |  |  |  |  |  |  |  |
| 6. Income (log10) | .39** | .35** | .43** | .17** | .11** | - |  |  |  |  |  |  |
| 7. Extraversion | -.08** | -.06** | .01 | -.05** | -.03 | .05** | - |  |  |  |  |  |
| 8. Agreeableness | -.10** | -.06** | .01 | -.15** | .09** | -.02 | .19** | - |  |  |  |  |
| 9. Conscientiousness | -.00 | -.01 | .11** | -.03* | .10** | .12** | .24** | .38** | - |  |  |  |
| 10. Neuroticism | -.07** | .02 | -.11** | -.15** | -.14** | -.12** | -.30** | -.37** | -.42** | - |  |  |
| 11. Openness | .12** | .08** | .19** | .07** | .03* | .03* | .31** | .18** | .20** | -.20** | - |  |
| 12. Income satisfaction | .18** | .11** | .21** | .10** | .15** | .43** | .08** | .06** | .15** | -.23** | -.03* | - |
| 13. Life satisfaction | .03* | -.00 | .08** | .03* | .09** | .23** | .18** | .16** | .22** | -.35** | .01 | .59** |

*Note.* Pearson's *r* indicated for all continuous variables, Spearman's rho used for gender ; 0 = female; 1 = male. *indicates *p* <.05, ** indicates *p* <.01.
