# Supplementary material for: Money matters (especially if you are good at math): Numeracy, verbal intelligence, education, and income in satisfaction judgments
Source: PLoS One. 2021 Nov 24;16(11):e0259331. doi: 10.1371/journal.pone.0259331 (PMC8612560; doi:10.1371/journal.pone.0259331)
Supplement: S2 Table — (DOCX) [file pone.0259331.s002.docx]

# Table S2. Linear regression analysis results of income (in thousands of $) predicted from objective numeracy, verbal logic, education, gender, age, age^2^, and Big-Five personality factors.

| Predictor | *beta* | *b* | *b*  95% CI  [LL, UL] | *p* | Fit |
| --- | --- | --- | --- | --- | --- |
| Intercept |  | 63.29 | [61.71, 64.87] | <.001 |  |
| Objective Numeracy | 0.18 | 4.06 | [ 3.43, 4.70 ] | <.001 |  |
| Verbal logic | 0.11 | 1.76 | [ 1.33, 2.18 ] | <.001 |  |
| Education | 0.33 | 13.31 | [12.31, 14.32] | <.001 |  |
| Gender | 0.17 | 7.32 | [ 5.24, 9.40 ] | <.001 |  |
| Age | 0.04 | 1.27 | [ 0.63, 1.90 ] | <.001 |  |
| Age^2^ | -0.12 | -1.94 | [-2.30, -1.59] | <.001 |  |
| Extraversion | 0.09 | 4.78 | [ 3.45, 6.12 ] | <.001 |  |
| Agreeableness | -0.07 | -4.73 | [-6.57, -2.88] | <.001 |  |
| Conscientiousness | 0.06 | 4.42 | [ 2.60, 6.24 ] | <.001 |  |
| Neuroticism | -0.05 | -2.59 | [-4.01, -1.17] | <.001 |  |
| Openness | -0.09 | -6.26 | [-7.91, -4.61] | <.001 |  |
|  |  |  |  |  | *R^2^*  = .31 |
|  |  |  |  |  | F(11,5513)=220.2, *p*<.001 |
|  |  |  |  |  | 95% CI[.29,.32] |
|  |  |  |  |  | Adjusted *R^2^=.*30 |
|  |  |  |  |  | AIC =55317 |
|  |  |  |  |  | BIC =55403 |
|  |  |  |  |  |  |

*Note.* *beta* indicates the standardized regression weights for continuous variables and partially standardized results for gender ; 0 = female; 1 = male. *b* represents unstandardized regression weights. *LL* and *UL* indicate the lower and upper limits of a confidence interval of the *b*, respectively.
