# Supplementary material for: Money matters (especially if you are good at math): Numeracy, verbal intelligence, education, and income in satisfaction judgments
Source: PLoS One. 2021 Nov 24;16(11):e0259331. doi: 10.1371/journal.pone.0259331 (PMC8612560; doi:10.1371/journal.pone.0259331)
Supplement: S3 Table — (DOCX) [file pone.0259331.s003.docx]

# Table S3. Regression analysis results of Income satisfaction and Life satisfaction predicted from objective numeracy, verbal logic, education, gender, age, age^2^, and the Big-Five personality factors.

|  | Income satisfaction | | | | |  | Life satisfaction | | | | |
| --- | --- | --- | --- | --- | --- | --- | --- | --- | --- | --- | --- |
|  | *beta* | *b* | *b*  95% CI  [LL, UL] | *p* | Fit |  | *beta* | *b* | *b*  95% CI  [LL, UL] | *p* | Fit |
| Intercept |  | 5.40 | [ 5.29, 5.50 ] | <.001 |  |  |  | 7.20 | [ 7.12, 7.28 ] | <.001 |  |
| Objective Numeracy | .10 | 0.14 | [ 0.09, 0.18 ] | <.001 |  |  | .02 | 0.02 | [-0.01, 0.05 ] | .183 |  |
| Verbal logic | .03 | 0.03 | [ 0.00, 0.06 ] | .029 |  |  | .03 | -0.00 | [-0.02, 0.02 ] | .760 |  |
| Education | .15 | 0.37 | [ 0.30, 0.44 ] | <.001 |  |  | .05 | 0.10 | [ 0.05, 0.15 ] | <.001 |  |
| Gender | .06 | 0.17 | [ 0.03, 0.31 ] | .019 |  |  | .06 | -0.02 | [-0.12, 0.08 ] | .700 |  |
| Age | .11 | 0.18 | [ 0.14, 0.23 ] | <.001 |  |  | .04 | 0.00 | [ 0.00, 0.01 ] | .003 |  |
| Age^2^ | .03 | 0.03 | [ 0.01, 0.06 ] | .009 |  |  | .06 | 0.04 | [ 0.02, 0.06 ] | <.001 |  |
| Extraversion | .07 | 0.22 | [ 0.13, 0.31 ] | <.001 |  |  | .11 | 0.26 | [ 0.19, 0.32 ] | <.001 |  |
| Agreeableness | -.02 | -0.09 | [-0.22, 0.04 ] | .159 |  |  | -.02 | 0.05 | [-0.04, 0.14 ] | .242 |  |
| Conscientiousness | .07 | 0.30 | [ 0.18, 0.43 ] | <.001 |  |  | .07 | 0.24 | [ 0.15, 0.33 ] | <.001 |  |
| Neuroticism | -.18 | -0.58 | [-0.67, -0.48] | <.001 |  |  | -.28 | -0.66 | [-0.72, -0.59] | <.001 |  |
| Openness | -.15 | -0.61 | [-0.73, -0.50] | <.001 |  |  | -.11 | -0.33 | [-0.41, -0.25] | <.001 |  |
|  |  |  |  |  | *R^2^*  = .13 |  |  |  |  |  | *R^2^*  = .15 |
|  |  |  |  |  | F(11,5543)=76.5, *p*<.001 |  |  |  |  |  | F(11,5513)=88.3, *p*<.001 |
|  |  |  |  |  | 95% CI[.12,.15] |  |  |  |  |  | 95% CI[.13,.17] |
|  |  |  |  |  | Adjusted *R^2^=.*13 |  |  |  |  |  | Adjusted *R^2^=.*15 |
|  |  |  |  |  | AIC = 25721 |  |  |  |  |  | AIC =21783 |
|  |  |  |  |  | BIC = 25807 |  |  |  |  |  | BIC =21869 |

*Note. beta* indicates the standardized regression weights for continuous variables and partially standardized results for Gender ; 0 = female; 1 = male. *b* represents unstandardized regression weights. *LL* and *UL* indicate the lower and upper limits of a confidence interval of the *b*, respectively.
