# Supplementary material for: Money matters (especially if you are good at math): Numeracy, verbal intelligence, education, and income in satisfaction judgments
Source: PLoS One. 2021 Nov 24;16(11):e0259331. doi: 10.1371/journal.pone.0259331 (PMC8612560; doi:10.1371/journal.pone.0259331)
Supplement: S4 Table — (DOCX) [file pone.0259331.s004.docx]

# Table S4. Regression analysis results of Income satisfaction and Life satisfaction predicted from income, objective numeracy, verbal logic, education, gender, age, age^2^, and the Big-Five personality factors.

|  | Income satisfaction | | | | |  | Life satisfaction | | | | |
| --- | --- | --- | --- | --- | --- | --- | --- | --- | --- | --- | --- |
|  | *beta* | *b* | *b*  95% CI  [LL, UL] | *p* | Fit |  | *beta* | *b* | *b*  95% CI  [LL, UL] | *p* | Fit |
| Intercept |  | 5.38 | [ 5.28, 5.48 ] | <.001 |  |  |  | 7.19 | [ 7.12, 7.27 ] | <.001 |  |
| Income (log_10_) | .39 | 2.46 | [ 2.28, 2.63 ] | <.001 |  |  | .23 | 1.01 | [ 0.88, 1.13 ] | <.001 |  |
| Objective Numeracy | .03 | 0.04 | [-0.00, 0.08 ] | .044 |  |  | -.02 | -0.02 | [-0.05, 0.01 ] | .198 |  |
| Verbal logic | -.03 | -0.03 | [-0.06, -0.00] | .020 |  |  | -.04 | -0.03 | [-0.05, -0.01] | .005 |  |
| Education | .04 | 0.09 | [ 0.02, 0.15 ] | .011 |  |  | -.01 | -0.02 | [-0.07, 0.03 ] | .434 |  |
| Gender | .00 | 0.01 | [-0.13, 0.14 ] | .958 |  |  | -.05 | -0.09 | [-0.19, 0.01 ] | .078 |  |
| Age | .07 | 0.12 | [ 0.08, 0.16 ] | <.001 |  |  | .01 | 0.02 | [-0.01, 0.05 ] | .207 |  |
| Age^2^ | .08 | 0.08 | [ 0.05, 0.10 ] | <.001 |  |  | .09 | 0.06 | [ 0.04, 0.08 ] | <.001 |  |
| Extraversion | .03 | 0.11 | [ 0.03, 0.20 ] | .015 |  |  | .09 | 0.21 | [ 0.15, 0.28 ] | <.001 |  |
| Agreeableness | -.01 | -0.04 | [-0.16, 0.08 ] | .499 |  |  | .02 | 0.07 | [-0.01, 0.16 ] | .094 |  |
| Conscientiousness | .04 | 0.18 | [ 0.07, 0.30 ] | .007 |  |  | .06 | 0.19 | [ 0.11, 0.28 ] | <.001 |  |
| Neuroticism | -.16 | -0.53 | [-0.62, -0.43] | <.001 |  |  | -.28 | -0.63 | [-0.70, -0.57] | <.001 |  |
| Openness | -.10 | -0.44 | [-0.54, -0.33] | <.001 |  |  | -.09 | -0.26 | [-0.34, -0.18] | <.001 |  |
|  |  |  |  |  | *R^2^* =.24 |  |  |  |  |  | *R^2^*  = .19 |
|  |  |  |  |  | F(12,5512)=144.1, *p*<.001 |  |  |  |  |  | F(12,5512)=104.6, *p*<.001 |
|  |  |  |  |  | 95% CI[.21,.25] |  |  |  |  |  | 95% CI[.17,.20] |
|  |  |  |  |  | Adjusted *R^2^=*.24 |  |  |  |  |  | Adjusted *R^2^=.18* |
|  |  |  |  |  | AIC = 25001 |  |  |  |  |  | AIC = 21549 |
|  |  |  |  |  | BIC = 25093 |  |  |  |  |  | BIC = 21642 |

*Note. beta* indicates the standardized regression weights for continuous variables and partially standardized results for Gender; 0 = female; 1 = male. *b* represents unstandardized regression weights. *LL* and *UL* indicate the lower and upper limits of a confidence interval of the *b*, respectively.
