# Supplementary material for: Money matters (especially if you are good at math): Numeracy, verbal intelligence, education, and income in satisfaction judgments
Source: PLoS One. 2021 Nov 24;16(11):e0259331. doi: 10.1371/journal.pone.0259331 (PMC8612560; doi:10.1371/journal.pone.0259331)
Supplement: S5 Table — (DOCX) [file pone.0259331.s005.docx]

# Table S5. Regression analysis results of Income satisfaction and Life satisfaction predicted from income, objective numeracy, verbal logic, education, gender, age, age^2^, and the Big-Five personality factors as well as the three interactions between income and objective numeracy, income and verbal logic and income and education.

|  | Income satisfaction | | | | |  | Life satisfaction | | | | |
| --- | --- | --- | --- | --- | --- | --- | --- | --- | --- | --- | --- |
|  | *beta* | *b* | *b*  95% CI  [LL, UL] | *p* | Fit |  | *beta* | *b* | *b*  95% CI  [LL, UL] | *p* | Fit |
| Intercept |  | 5.31 | [ 5.16, 5.47 ] | <.001 |  |  |  | 7.06 | [ 6.95, 7.18 ] | <.001 |  |
| Income (log_10_) | .45 | 2.73 | [ 2.53, 2.93 ] | <.001 |  |  | .25 | 1.11 | [ 0.96, 1.26 ] | <.001 |  |
| Objective Numeracy | .01 | 0.02 | [-0.03, 0.06 ] | .400 |  |  | -.03 | -0.03 | [-0.06, 0.00 ] | .078 |  |
| Verbal logic | .00 | 0.00 | [-0.03, 0.03 ] | .919 |  |  | -.00 | -0.00 | [-0.03, 0.02 ] | .789 |  |
| Education | .01 | 0.03 | [-0.05, 0.10 ] | <.507 |  |  | -.02 | -0.04 | [-0.10, 0.02 ] | .164 |  |
| Gender | -.01 | -0.04 | [-0.19, 0.11 ] | .606 |  |  | -.06 | -0.11 | [-0.22, -0.00] | .048 |  |
| Household member | -.04 | -0.07 | [-0.12, -0.02] | .008 |  |  | .01 | 0.01 | [-0.02, 0.05 ] | .491 |  |
| Age | .05 | 0.11 | [ 0.04, 0.18 ] | .002 |  |  | .01 | 0.01 | [-0.04, 0.06 ] | .754 |  |
| Age^2^ | .06 | 0.09 | [ 0.04, 0.13 ] | <.001 |  |  | .06 | 0.06 | [ 0.02, 0.09 ] | <.001 |  |
| Extraversion | .03 | 0.12 | [ 0.02, 0.21 ] | .015 |  |  | .10 | 0.23 | [ 0.16, 0.30] | <.001 |  |
| Agreeableness | -.00 | -0.01 | [-0.14, 0.12 ] | .899 |  |  | .03 | 0.09 | [-0.01, 0.19 ] | .069 |  |
| Conscientiousness | .04 | 0.19 | [ 0.07, 0.32 ] | .003 |  |  | .06 | 0.19 | [ 0.10, 0.29 ] | <.001 |  |
| Neuroticism | -.16 | -0.52 | [-0.62, -0.42] | <.001 |  |  | -.27 | -0.61 | [-0.69, -0.54] | <.001 |  |
| Openness | -.11 | -0.46 | [-0.57, -0.34] | <.001 |  |  | -.09 | -0.27 | [-0.36, -0.19] | <.001 |  |
| Income (log_10_) x Objective numeracy | .06 | 0.19 | [0.09, 0.29] | < .001 |  |  | .05 | 0.10 | [ 0.03, 0.18 ] | .007 |  |
| Income (log_10_) x Verbal logic | .03 | 0.08 | [0.01, 0.14] | .017 |  |  | .04 | 0.07 | [ 0.02, 0.11 ] | .006 |  |
| Income (log_10_) x Education | .07 | 0.38 | [0.21, 0.54] | < .001 |  |  | .04 | 0.16 | [ 0.03, 0.28 ] | .012 |  |
|  |  |  |  |  | *R^2^*  = .24 |  |  |  |  |  | *R^2^*  = .18 |
|  |  |  |  |  | F(16,4573)=90.6, *p*<.001 |  |  |  |  |  | F(16,4573)=62.7, *p*<.001 |
|  |  |  |  |  | 95% CI[.22,.26] |  |  |  |  |  | 95% CI[.16,.20] |
|  |  |  |  |  | Adjusted *R^2^=.*24 |  |  |  |  |  | Adjusted *R^2^=.*18 |
|  |  |  |  |  | AIC =20737 |  |  |  |  |  | AIC =17997 |
|  |  |  |  |  | BIC =20853 |  |  |  |  |  | BIC =18113 |

*Note. beta* indicates the standardized regression weights for continuous variables and partially standardized results for Gender ; 0 = female; 1 = male. *b* represents unstandardized regression weights. *LL* and *UL* indicate the lower and upper limits of a confidence interval of the *b*, respectively.
