# Supplementary material for: Money matters (especially if you are good at math): Numeracy, verbal intelligence, education, and income in satisfaction judgments
Source: PLoS One. 2021 Nov 24;16(11):e0259331. doi: 10.1371/journal.pone.0259331 (PMC8612560; doi:10.1371/journal.pone.0259331)
Supplement: S6 Table — (DOCX) [file pone.0259331.s006.docx]

# Table S6. Regression analysis results of Income satisfaction and Life satisfaction predicted from income, objective numeracy, verbal logic, education (as a factor), gender, age, age^2^, and the Big-Five personality factors as well as the three interactions between income and objective numeracy, income and verbal logic and income and education.

|  | Income satisfaction | | | |  | Life satisfaction | | | |
| --- | --- | --- | --- | --- | --- | --- | --- | --- | --- |
|  | *b* | *b*  95% CI  [LL, UL] | *p* | Fit |  | *b* | *b*  95% CI  [LL, UL] | *p* | Fit |
| Intercept | 5.04 | [ 4.63, 5.44 ] | <.001 |  |  | 7.08 | [6.78, 7.38] | <.001 |  |
| Income (log_10_) | 2.07 | [ 1.46, 2.68 ] | <.001 |  |  | 0.73 | [0.28, 1.18] | <.001 |  |
| Objective Numeracy | 0.02 | [-0.02, 0.07 ] | .345 |  |  | -0.03 | [-0.07, 0.00] | .053 |  |
| Verbal logic | 0.00 | [-0.03, 0.03 ] | .865 |  |  | -0.00 | [-0.03, 0.02] | .851 |  |
| Education 1 | 0.45 | [ 0.04, 0.86 ] | .289 |  |  | 0.13 | [-0.18, 0.43] | .572 |  |
| Education 2 | 0.21 | [-0.18, 0.60 ] | .018 |  |  | -0.08 | [-0.37, 0.21] | .005 |  |
| Education 4 | 0.18 | [-0.23, 0.60 ] | .784 |  |  | -0.02 | [-0.32, 0.29] | .374 |  |
| Education 5 | 0.49 | [ 0.02, 0.95 ] | .065 |  |  | 0.08 | [-0.27, 0.42] | .149 |  |
| Gender | -0.05 | [-0.20, 0.10 ] | .508 |  |  | -0.12 | [-0.23, -0.01] | .036 |  |
| Household member | -0.06 | [-0.11, -0.01] | .013 |  |  | 0.02 | [-0.02, 0.05] | .400 |  |
| Age | 0.11 | [ 0.04, 0.18 ] | .002 |  |  | 0.01 | [-0.04, 0.06] | .756 |  |
| Age^2^ | 0.09 | [ 0.04, 0.13 ] | <.001 |  |  | 0.06 | [0.02, 0.09] | <.001 |  |
| Extraversion | 0.12 | [ 0.02, 0.21 ] | .013 |  |  | 0.23 | [0.16, 0.30] | <.001 |  |
| Agreeableness | -0.01 | [-0.14, 0.12 ] | .908 |  |  | 0.09 | [-0.01, 0.19] | .069 |  |
| Conscientiousness | 0.18 | [ 0.05, 0.31 ] | .005 |  |  | 0.19 | [0.10, 0.29] | <.001 |  |
| Neuroticism | -0.53 | [-0.63, -0.43] | <.001 |  |  | -0.62 | [-0.69, -0.54] | <.001 |  |
| Openness | -0.45 | [-0.57, -0.34] | <.001 |  |  | -0.27 | [-0.36, -0.19] | <.001 |  |
| Income (log_10_) x Objective numeracy | 0.18 | [ 0.08, 0.29] | <.001 |  |  | 0.12 | [0.04, 0.20] | .002 |  |
| Income (log_10_) x Verbal logic | 0.08 | [ 0.02, 0.15] | .013 |  |  | 0.06 | [0.01, 0.11] | .010 |  |
| Income (log_10_) x Education 1 | 0.05 | [-0.62, 0.71] | .071 |  |  | 0.13 | [-0.36, 0.62] | .025 |  |
| Income (log_10_) x Education 2 | 0.59 | [-0.05, 1.24] | .014 |  |  | 0.55 | [0.07, 1.03] | .012 |  |
| Income (log_10_) x Education 4 | 1.18 | [ 0.42, 1.94] | .027 |  |  | 0.32 | [-0.24, 0.88] | .259 |  |
| Income (log_10_) x Education 5 | 1.13 | [ 0.20, 2.07] | .157 |  |  | 0.29 | [-0.40, 0.99] | .364 |  |
|  |  |  |  | *R^2^*  = .24 |  |  |  |  | *R^2^*  = .18 |
|  |  |  |  | *F(22,4567)=66.9, p<.001* |  |  |  |  | *F(22,4567)=46.4, p<.001* |
|  |  |  |  | 95% CI[.22,.26] |  |  |  |  | 95% CI[.16,.20] |
|  |  |  |  | Adjusted *R^2^=.*24 |  |  |  |  | Adjusted *R^2^=.*18 |
|  |  |  |  | AIC =20731 |  |  |  |  | AIC =17994 |
|  |  |  |  | BIC =20885 |  |  |  |  | BIC =18148 |

*Note. b* represents unstandardized regression weights Gender ; 0 = female; 1 = male, Education; 1 = Less than High School diploma; 2 = High school diploma; 3 = Some college or Associates degree; 4 = Bachelor’s degree; 5 = Master’s degree or more, centered on 3 . *LL* and *UL* indicate the lower and upper limits of a confidence interval of the *b*, respectively. No *beta* (standardized regression weights) calculated.
